# Factors influencing the adoption of innovation in spine surgery: An international survey of AO spine network

**DOI:** 10.1016/j.bas.2025.104206

**Published:** 2025-02-08

**Authors:** Arun Kumar Viswanadha, Luca Ambrosio, Pieter-Paul A. Vergroesen, Zorica Buser, Hans Joerg Meisel, Nancy Santesso, Jason P.Y. Cheung, Yabin Wu, Hai V. Le, Gianluca Vadalà, Amit Jain, Andreas K. Demetriades, Sam K. Cho, Patrick C. Hsieh, Ashish Diwan, Tim Yoon, Sathish Muthu, AO Spine Knowledge Forum Degenerative

**Affiliations:** a Reva Spine Centre, Visakhapatnam, India; b Operative Research Unit of Orthopaedic and Trauma Surgery, Fondazione Policlinico Universitario Campus Bio-Medico, Rome, Italy; c Research Unit of Orthopaedic and Trauma Surgery, Department of Medicine and Surgery, Università Campus Bio-Medico di Roma, Rome, Italy; d Department of Orthopaedics, Noordwest Hospitals, Alkmaar, the Netherlands; e Gerling Institute, New York City, NY, USA; f Department of Neurosurgery, BG Klinikum Bergmannstrost Halle, Halle, Germany; g Department of Health Research Methods Evidence and Impact, McMaster University, Hamilton, ON, Canada; h Department of Orthopaedics and Traumatology, The University of Hong Kong, China; i AO Spine International, AO Foundation, Davos, Switzerland; j Department of Orthopaedic Surgery, University of California, Davis, CA, USA; k Department of Orthopaedic Surgery, Johns Hopkins University, Baltimore, MD, USA; l Department of Neurosurgery, Edinburgh Spinal Surgery Outcome Studies Group, Royal Infirmary Edinburgh, Edinburgh, UK; m Department of Orthopedic Surgery, Icahn School of Medicine at Mount Sinai, New York, NY, USA; n Department of Neurological Surgery, Keck School of Medicine, University of Southern California, CA, USA; o Spine Labs, St George and Sutherland Clinical School, University of New South Wales, Kogarah, NSW, Australia; p Spine Service, Department of Orthopaedic Surgery, St George and Sutherland Clinical School, University of New South Wales, Kogarah, NSW, Australia; q Department of Orthopaedics, Emory University, Atlanta, GA, USA; r Department of Orthopedics, Government Karur Medical College and Hospital, Karur, Tamil Nadu, India; s Department of Spine Surgery, Orthopedic Research Group, Coimbatore, Tamil Nadu, India; t Department of Biotechnology, Faculty of Engineering, Karpagam Academy of Higher Education, Coimbatore, Tamil Nadu, India

**Keywords:** Practice pattern, Adoption cycle, Spine surgery, Challenges

## Abstract

**Introduction:**

Knowledge translation from research to clinical practice can often be challenging, and practice modification patterns among surgeons may stem from a variety of sources, including personal experience, peer influence, ongoing education, and evolving research findings.

**Research question:**

This study aimed to investigate the adoption patterns amongst surgeons for newer innovations and to analyse the factors affecting the implementation of the same in clinical practice. We used the adoption of osteobiologics as a case example.

**Methods:**

An international expert survey was conducted among AO Spine users and members. The survey, comprising 30 items, explored surgeons' demographics, risk aversion, and factors influencing practice change. We categorized the innovation-adoptive nature of the surgeons and scored their risk-adoptive behaviour.

**Results:**

A total of 458 responses were received from surgeons across 81 countries including 433 male (95%), orthopaedic surgeons (n = 263; 57%) from university-affiliated hospitals (n = 185; 40%). Most were in the early majority phase of the innovation-adoption cycle (n = 174; 38%) with a majority in the ‘high-moderate’ risk-adoption category (n = 396; 86%). This risk adoption behaviour had a significant correlation with their appetite for innovation (r = 0.182,p=<0.001). About 67.9% of respondents preferred scientific literature and conference presentations showcasing solid clinical evidence to be the most influential factor in driving change in their clinical practice. Material logistics (55%) is considered an important barrier to practice modification followed by familiarity (50%) and financial reimbursements (25%).

**Discussion & conclusion:**

A complex interplay exists between risk-adoptive behaviour amongst surgeons and the factors influencing a change in their clinical practice. Although most surgeons were in the early adoptive phase in accepting the innovations into their clinical practice, they were also equally noted to be risk tolerant. Hence, a successful adoption of practice-changing innovation hinges on addressing not only logistical and financial challenges but also on providing robust scientific evidence to drive the necessary change in clinical practice.

## Highlights

• Most spine surgeons are in the early majority phase of innovation-adoption with moderate risk-adoption behaviour.
• Risk adoption behaviour significantly correlates with their appetite for innovation.
• Most surgeons find scientific literature and conference presentations with solid clinical evidence most influential.
• Material logistics is a key barrier to practice modification, followed by familiarity and financial reimbursements.
• A complex interplay exists between risk-adoptive behavior among surgeons and factors influencing clinical practice change.

## 1 Introduction

Surgeons, like professionals in any field, exhibit a spectrum of adaptive behaviours and practice modification patterns shaped by a multitude of factors. Knowledge translation from research to clinical practice can often be challenging, and practice modification patterns among surgeons may stem from a variety of sources, including personal experience, peer influence, ongoing education, and evolving research findings.

Osteobiologics are increasingly adopted in spinal surgery (Ahn et al., 2011). In recent years, the integration of osteobiologics into anterior cervical discectomy and fusion (ACDF) procedures has become increasingly common, driven by advancements in biomaterials and a deeper understanding of bone biology (Arun-Kumar et al., 2024; Hamouda et al., 2024). The AO Spine knowledge forum has recently published a guideline document *AOGO – AO guideline for the use of Osteobiologic in ACDF surgery* (Meisel et al., 2024). The aim of this study is to understand factors that influence spinal surgeon adoption of innovations such as osteobiologics. For this study, we used the AOGO guideline for the use of osteobiologics as a case example to ascertain surgeon attitudes. We sought to examine opinions through psychometric evaluation of their behaviour to implementation of osteobiologics put forth by professional societies.

## 2 Methodology:

*Study participants:* An English-language 30-item survey was designed to examine the current practices and their practice modification behaviour of spine surgeons with regard to the evidence surrounding osteobiologics in ACDF surgery. An e-mail was sent to all the members of AO Spine on a web-based platform requesting them to participate in the survey. The link was made available for 30 days with two reminders sent out during the period.

*Study questionnaire:* Participants’ country and AO Spine region of practice, gender, age, years of practice in spine surgery, speciality, caseload, practice setting and information about spine surgery fellowship were retrieved.

The surgeons were also asked to define their innovation-adoption attitude into either of the following five categories as described by EM Rogers (2003): innovator who distinguishes oneself by venturesomeness, tolerance of risk, fascination with novelty and willingness to 'leave the village' to learn; early adopter who is a self-conscious experimenter who tries out selective ideas to explore its usefulness; early majority who is more risk-averse than the groups above who is readier to hear about innovations relevant to personal practice and to try innovations that meet the immediate needs; late majority who adopts an innovation when it appears to be the new status quo or standard of practice, not before; laggard, whose reference is the tried and tested, who considers nothing is more reliable than what has worked in their hands in the past and hesitant to change practice (Berwick, 2003).

The risk-adoption behaviour scale developed by Pearson in 1995 was used to measure the risk-taking attitudes of surgeons (Pearson et al., 1995). This scale was developed to assess how likely physicians are to engage in or avoid risky behaviours in their professional practice. It consists of six questions asked on a six-point Likert scale from ‘strongly disagree’ to ‘strongly agree’ rated from 1 to 6 respectively resulting in the minimum possible score of 6 to a maximum score of 36 for every respondent answering all 6 questions. They were further categorized into ‘high’ (28–36), ‘moderate’ (21–27), ‘low’ (14–20) or ‘very-low’ (6–13) risk-adoption categories based on their scores.

## 3 Statistical analysis

Continuous variables were presented as mean and standard deviation while categorical variables were presented as frequencies and percentages. We used Cronbach's alpha to measure the validity of the scale utilized to measure the risk-adoption behaviour. All possible independent variables collected through the survey were analyzed using univariate regression by forward entry and retained in the final model when p < 0.1. Correlation analysis and multivariable analyses were performed to identify factors affecting the innovation-adoption category and risk adoption score by Pearson correlation and multivariate logistic regression analysis respectively. A two-tailed p-value less than 0.05 was considered statistically significant. All statistical analysis was performed in SPSS version 25 (IBM Corp, Armonk, USA).

## 4 Results

### 4.1 Demography of participants

**Fig. 1:**
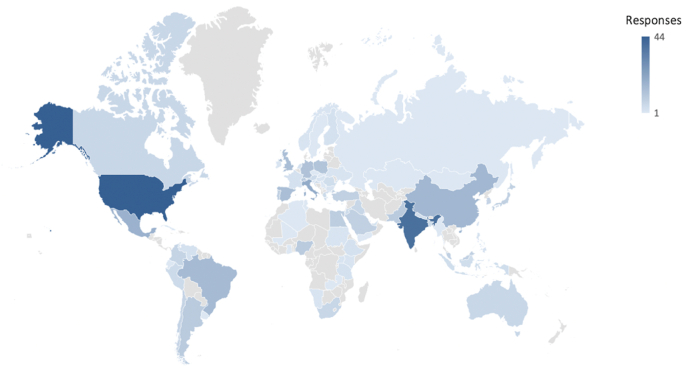
Heatmap showing geographic distribution of participants (n = 458).

A total of 458 responses were received from AO Spine members from 81 different countries across the globe. Most of the responses were recorded from Europe and Southern Africa region (n = 161; 35.2%) followed by Asia Pacific (n = 116; 26.7%), Latin America (n = 66; 14.4%), Middle East and Northern Africa (n = 62; 13.5%) and North America (n = 56; 12.2%) as shown in Fig. 1.

Out of all respondents, 433 were male (94.5%) and 21 were female (4.6%). Most respondents were orthopaedic surgeons (n = 263; 57.4%), followed by neurosurgeons (n = 186; 40.6%) and other spine care professionals (n = 9; 2.0%). Regarding institutional affiliation, 40.4% were practicing in a university-affiliated hospital (n = 185), while 28.4% were private practitioners (n = 130) and 27.5% were affiliated with a government hospital (n = 126). A notable majority, 365 of 458 respondents, were working in urban areas (79.7%) followed by 79 in Suburban regions (17.2%) and only 14 in rural areas (3.0%). Notably 65% of respondents had completed a structured spine surgery fellowship while 34.9% were not exposed to any spine fellowship program. Only 98 respondents had less than 5 years of work experience whereas 57 had more than 20 years of work experience after their fellowship. There were 123 participants (26.9%) working at low-volume institutions (<100 spine surgeries/year), 166 at middle-volume institutions (100–200 surgeries/year) and 169 working at high-volume institutions (>200 surgeries/year). Detailed characteristics of the participants are given in Table 1.

**Table 1:** Demographics of survey respondents.

Characteristics of Participants	Overall (n = 458)
*Specialty*	
Orthopedics	263 (57.4%)
Neurosurgery	186 (40.6%)
Others	9 (1.97%)
***Hospital Setting***	
University-affiliated hospital	185 (40.4%
Private	130 (28.4%)
Government Hospital	126 (27.5%)
Others	17 (3.7%)
***Age Categories***	
25–34	50 (10.9%)
35–44	179 (39.0%)
45–54	135 (29.5%)
55–64	62 (13.5%)
65+	32 (7.0%)
***Years in Practice***	
<5 years	93 (20.3%)
5–10 years	115 (25.1%)
11–15	79 (17.3%)
16–20	57 (12.4%)
>20 years	114 (24.9%)
***Number of ACDF surgeries performed in a year***	
1–20	206 (45.0%)
21–50	168 (36.7%)
51–100	67 (14.6%)
>100	17 (3.7%)

### 4.2 Innovation categorization

**Fig. 2:**
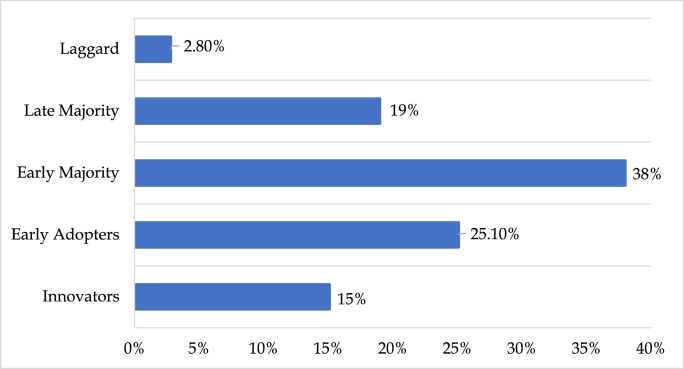
Innovation-adoption categorization of the respondents.

Of all the respondents, 15.0% were categorized as “Innovators” who were fascinated with novelty and were willing to venture into newer procedures while 25.1% described themselves as “Early Adopters” who were self-conscious experimenters and would like to try out selective ideas to explore its usefulness; 38.0% were “Early majority” who were ready to hear about innovations relevant to their clinical practice and try innovations that meet immediate needs, and 19.0% were “Late majority” who adopt innovations when they become standard of practice and 2.8% described themselves as “Laggard”, who were hesitant to change their standard of practice as shown in Fig. 2.

Of all respondents, neurosurgeons were significantly categorized as innovators (r = 0.165, p = 0.01). Surgeons working at university-affiliated hospitals tended to adopt newer techniques when compared to private practitioners (r = 0.097, p = 0.037). Innovation categorization was also directly proportional to the caseload of the surgeons, with a higher number of operations performed being associated with the category of innovators and early adopters (r = 0.138, p = 0.003), and was open to newer innovations into clinical practice. Regression analysis of the demographic factors that contributed to the innovation categorization of the surgeons revealed being a neurosurgeon significantly attributed to the respondent innovation-adoption categorization (β = 0.149, p = 0.003).

### 4.3 Risk adoption behaviour

The scale utilized for risk-adoption behaviour demonstrated a Cronbach's alpha of 0.71 which demonstrated an acceptable level of validity for its utilization in the study. While only 27.1% of respondents strongly agreed or agreed that they enjoyed taking risks, a significant 44.76% proportion of the respondents tried to avoid uncertain outcomes. Additionally, 34.1% agreed that taking risks did not bother them if high gains were involved, suggesting a situational willingness to take risks. Security was a clear priority, with 77.5% considering it an important element in every aspect of their lives. The perception of others also played a role, as 28.9% of respondents had been told they seemed to enjoy taking chances. Lastly, 52.0% rarely took risks when alternatives existed, indicating a general preference for safer choices.

**Fig. 3:**
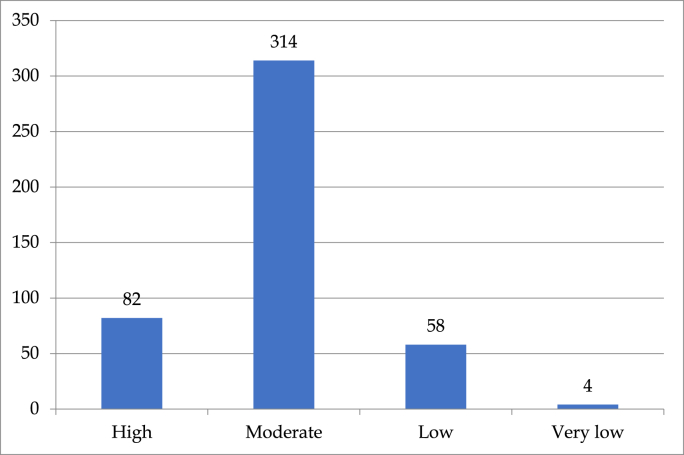
Categorization of respondents about their risk adoption behaviour.

Overall, the data portrays a higher risk adaptive behaviour among the surgeons with a preference towards preemptive implementation of innovation in clinical care. When the respondents were grouped into 4 categories based on their risk-adoption score, 82 respondents fell into the ‘*high risk’* group while 314, 58, and 4 respondents belonged to ‘*moderate risk’,* ‘*low risk’*, and ‘*very low risk*” group respectively, as shown in Fig. 3. Around 86% of the respondents belonged to the ‘high-moderate’ risk category. Risk-adoption behaviour has shown a significant direct correlation with the innovation-categorization of the participants (r = 0.182, p = 0.01) demonstrating that higher risk-taking group respondents tended to adopt newer techniques or guidelines faster compared to others. The risk adoption score had no significant correlation with any other demographic factors such as region, age, sex, speciality, area of practice, or caseload. Similarly, on regression analysis, none of the demographic factors was significantly associated with the risk-adoption behaviour of the participants.

### 4.4 Drivers of practice change

In response to the question, “What would persuade you to change your practice to adopt new guidelines?" 458 participants were asked to rank four factors from 1 (most important) to 5 (not important). The factors included scientific literature or conference presentations showing solid clinical evidence, recommendations by globally recognized experts, personal familiarity, and advice from mentors. The results indicate that scientific literature or conference presentations were the most influential, with 67.9% of participants ranking it as the most important factor (score of 1). This option had a mean rank of 1.5, reflecting its strong influence. Recommendations from globally recognized experts were the second most persuasive factor, with 19.2% ranking it as the most important and 52.8% as the second most important, resulting in a mean rank of 2.2. Personal familiarity and advice from mentors were less influential, with mean ranks of 3.2. Personal familiarity had a significant portion of participants (42.4%) rating it as least important (score of 5). Similarly, 41.3% rated mentors as the least important factor. Overall, the data highlights that solid clinical evidence from the scientific literature is the primary driver for changing clinical practice, while personal and mentor-related factors are considered less compelling.

**Fig. 4:**
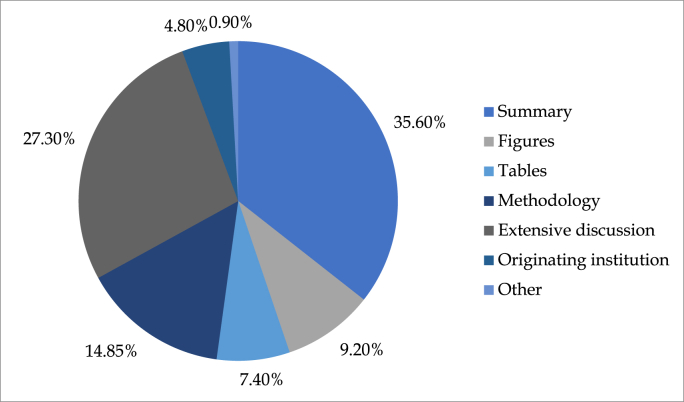
Key sections of guidelines that surgeons prefer for practice modification.

When asked about what is most useful when guidelines are published, 35.6% (n = 163) of respondents chose “the summary”, followed by 27.3% (n = 125) who felt that the extensive discussion part on how recommendations were made, whereas 14.9% (n = 68) said that the methodology part was most helpful. Fewer proportions at 9.2% (n = 42) and 7.4% (n = 34) preferred looking for figures and tables in a guideline. Only 4.8% (n = 22) said that the originating institution producing the guideline was helpful (see Fig. 4).

### 4.5 Limiting factors for practice modification

**Fig. 5:**
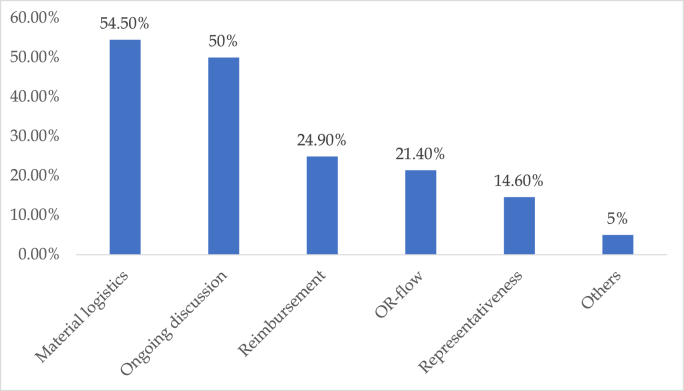
Limiting factors for change in practice to adopt new guidelines.

“Material logistics” was the most significant limiting factor for changing one's practice to adopt new guidelines, with 54.6% of respondents indicating it as a concern. The second most identified limiting factor, affecting 50.0% of respondents was “ongoing discussion surrounding the topic", and “reimbursement" was another notable concern, highlighted by 24.9% of participants. “OR-flow" and “representatives" were considered limiting factors by 21.4% and 14.6% of respondents, respectively. Overall, the data suggests that logistical and financial challenges, along with the need for ongoing discussion, are the primary barriers to adopting new guidelines as shown in Fig. 5.

## 5 Discussion

The results of this international expert survey shed light on the complex landscape of surgical practice modification patterns of spine surgeons in the face of new guidelines on innovations that are being published. The discussion herein delves into the nuances revealed by the survey findings, offering insights into the factors influencing surgeons' attitudes, behaviours, and decision-making processes.

Firstly, the demographic distribution of survey participants reflects a diverse representation across various regions and professional backgrounds. The majority of respondents were male orthopaedic surgeons and neurosurgeons, predominantly practising in urban settings and affiliated with high-volume institutions. One notable finding is the disparity in awareness and training regarding evidence among respondents. Despite the growing body of literature and guidelines, a significant proportion of surgeons reported limited formal training in this domain. This highlights a potential gap in educational initiatives and underscores the importance of continuing education to ensure evidence-based practice and optimal patient care. Previous studies have shown that large conferences and courses, and educational outreach with experts or trained operators yielded positive effects on changing practice (Davis et al., 1995, Gurwitz et al., 1990, Hulscher et al., 1999, Oxman et al., 1995, Salzmann et al., 2017).

The survey results also provide insights into surgeons' risk-taking behaviour and attitudes towards innovation. The categorization of respondents into distinct groups, such as innovators, early adopters, and late majority, offers a nuanced understanding of the spectrum of risk aversion within the surgical community (Berwick, 2003). Interestingly, while a considerable proportion of respondents expressed a preference for security and aversion to uncertain outcomes, there was also a notable willingness to embrace innovation, particularly when supported by solid clinical evidence. Although 27.1% of the respondents reported enjoying taking risks, one must realize that the driver is not only on the part of the surgeon but also on the patients who demand the recent innovation to be a part of their treatment process. Hence, this process of innovation adoption is a combined shared decision between the surgeon and the patients having discussed both the advantages and disadvantages of all the options available for a given condition. This risk appetite behaviour found among the patients and surgeons not only involves the adoption of recent innovation into the care process but also increases healthcare costs risking the lack of long-term efficacy and safety. This is also reflected in the risk-adoption behaviour categorization of the respondents where 86% belonged to the ‘high-moderate’ risk category.

The discussion on barriers to practice modification reveals multifaceted challenges, with material logistics, ongoing discussions, and reimbursement considerations emerging as prominent concerns. These logistical and financial hurdles underscore the complex interplay between clinical practice, institutional dynamics, and external factors shaping surgeons' decision-making processes. The barriers to change practice can arise at different stages in the health care system i.e. at the level of the patient, at practicing individual, at health care team and organization (Pippalla et al., 1995; Grol, 2001; Davis et al., 1995; Davis et al., 1999). Addressing these barriers requires a collaborative effort involving stakeholders at various levels to facilitate the implementation of evidence-based guidelines and promote best practices in patient care (Grol, 2001).

Furthermore, the survey findings elucidate the influential factors driving practice change among surgeons. Scientific literature and conference presentations emerge as the primary drivers, underscoring the importance of robust clinical evidence in shaping clinical practice. Recommendations from globally recognized experts also wield significant influence, highlighting the role of peer endorsement and expert consensus in guiding surgical decision-making. Although there is a steady increase in the introduction of newer implants in the spine implant market, their superiority over the existing implants is not strongly demonstrated. Usually, the newer implants demonstrate only ‘non-inferiority’ rather than ‘superiority’ to the existing implants before they are introduced into the market while obtaining a market distribution license from authorities. Further, most of the studies favoring their non-inferiority are industry-sponsored thereby limiting their objectivity (Mauri and D'Agostino, 2017). Hence, to determine the unbiased effectiveness of the introduced implants, it usually takes a couple of years following its introduction. This explains why the majority of the surgeons were in the early majority category when it comes to innovation adoption with moderate risk appetite as noted in this survey. However, we noted a considerable amount of surgeons categorized themselves as innovators (15%) and early adopters (25.1%) with high-risk appetites. The risk inherent to this behaviour and aptitude cannot be ignored. Considering the risk involved with this adoptive behaviour, informed decision with the patient remains paramount.

In conclusion, this survey offers a comprehensive exploration of surgeons' practice modification patterns and attitudes towards the implementation of innovations in clinical practice. By elucidating the underlying factors influencing surgical decision-making, these findings provide valuable insights for guiding educational initiatives, policy development, and future research endeavours aimed at optimizing patient outcomes and advancing the field of spinal surgery. Future educational initiatives could be designed to evaluate the effectiveness and safety of the innovations in the field to aid in modifying its adoption pattern. Further, policy measures to ensure its availability and accessibility to the patients would strengthen the clinical utility of these innovations.

## 6 Limitations

While the survey provides valuable insights into surgeons' attitudes and factors that drive practice modification, several limitations should be acknowledged. Firstly, the study's reliance on self-reported data introduces the potential for response bias and inaccuracies, as respondents may provide socially desirable or incomplete responses. Additionally, the survey sample primarily comprises AO Spine members, which may not fully represent the broader population of spine surgeons worldwide, limiting the generalizability of the findings. Furthermore, the survey's design may not capture the full spectrum of factors influencing surgeons' decision-making processes, such as institutional policies, patient preferences, or economic considerations. Moreover, the survey's cross-sectional nature precludes the assessment of temporal trends or causal relationships, necessitating caution in interpreting the observed associations. Lastly, the survey's focus on surgeons' perspectives may overlook the perspectives of other stakeholders, such as patients, allied healthcare professionals, or policymakers, which could provide valuable insights into the broader implications of the decision making process. Future research efforts should aim to address these limitations to provide a more comprehensive understanding of the factors shaping surgical practice in this domain.

## 7 Conclusion

In summary, this survey among AO Spine members illuminates the intricate dynamics shaping surgeons' approaches to incorporating innovations in clinical practice. An increasing number of surgeons were in the early majority phase to adopt the innovations with ‘high-moderate’ risk-adoption category. Considering the risk involved with this adoptive behaviour, informed decision with the patient remains paramount. When it comes to convincing respondents to adopt innovations into clinical practice, scientific literature and conference presentations featuring strong clinical evidence are the most persuasive. Successful adoption of innovation depends on overcoming logistical and financial hurdles while presenting strong scientific evidence to justify changes. Future strategies should cater to the risk-adoptive tendencies of respondents by emphasizing clear, evidence-based benefits from secured regulated data sources such as data registries.

## Ethical approval and informed consent statement

No formal institutional review board approval was required for this study. All the participants signed a digital informed consent and agreed on the use of their anonymized responses for research purposes.

Data availability statement

The data generated and analyzed during this study will be made available upon reasonable request.

## Funding statement

This study was organized and funded by AO Spine through the AO Spine Knowledge Forum Degenerative, a focused group of international spine degeneration experts. AO Spine is a clinical division of the AO Foundation, which is an independent medically-guided not-for-profit organization. Study support was provided directly through the AO Spine Research Department.

## Declaration of competing interest

The authors declare that they have no known competing financial interests or personal relationships that could have appeared to influence the work reported in this paper.
